# Nutrition Capacity Building to Meet National Priorities: Lessons Learned in Developing and Implementing Malawi's First Dietetics Program

**DOI:** 10.9745/GHSP-D-20-00687

**Published:** 2021-12-31

**Authors:** Sanele Nkomani, Lynne M. Ausman, Elizabeth Marino-Costello, Bernadette Chimera, Alexander Kalimbira, Agnes Mwangwela, Molly Uebele-Harrigan, John Phuka, Shibani Ghosh

**Affiliations:** a Tufts University, Boston, MA, USA.; b Kamuzu University of Health Sciences, Blantyre, Malawi.; c Lilongwe University of Agriculture and Natural Resources, Lilongwe, Malawi.

## Abstract

The current nutrition situation in Malawi, characterized by high rates of malnutrition in communities and hospitals and a rapidly increasing burden of overweight/obesity and diet-related noncommunicable diseases, highlights the urgent need for registered dietitians, who have a proven track record in the prevention and management of all forms of malnutrition and improving patient outcomes. However, dietetics practice has been described as underdeveloped and fragmented in many parts of Africa, exacerbated by a severe and chronic shortage of dietetics professionals and a lack of nutrition and dietetic education programs in most African countries.

We share early lessons learned in the development and implementation of the first dietetics program in Malawi. Within 6 years, the program produced 10 graduate dietitians who have filled the first clinical dietitian posts in Malawian public hospitals. This early success can be attributed to the model used to develop and implement the program, which included early stakeholder engagement to define the priority skills and competencies of a Malawian dietitian, the use of internationally recognized training standards, and the development of strategic institutional partnerships that brought together complementary skills and expertise. Furthermore, using existing resources and recruiting students with a nutrition and health background accelerated implementation. The current dietetics curriculum responds to the national nutrition and health policy direction and strategic objectives. Early and sustained government engagement was crucial in creating demand and securing career prospects for graduates. Although still in its infancy, dietitians in Malawi are poised to contribute significantly to alleviating the country's complex nutrition challenges.

## Abstract (precis)

We describe the lessons learned in building nutrition capacity through the development and implementation of the first dietetics training program in Malawi.

## Key Messages

- We developed the first-ever clinical dietetics program in Malawi to equip students with knowledge, attitudes, and skills in nutrition support, health promotion, and disease prevention at the community and health facility levels.
- We addressed challenges in curriculum implementation, lack of supervision, and lack of capacity-building resources by using unique approaches and building partnerships with existing programs and professionals.
- Building a dietitian workforce to serve the needs of the population requires a commitment to funding the program, building research capacity, and developing regulatory and practice guidelines.

## INTRODUCTION

Registered dietitians are recognized globally as being essential to delivering preventative and curative health services and integral members of multidisciplinary health care teams.^1^^,^^2^ A dietitian's skills and competencies rest on their ability to interpret and effectively communicate complex theoretical nutrition knowledge to health care providers, policy makers, communities, and individuals, to promote and maintain health across the lifespan and in different disease states.^3^ Dietetic interventions have shown to be highly efficacious in improving health outcomes and reducing costs for numerous conditions at all levels of health care.^3^^–^^6^

Despite a globally observed exponential growth in the dietetics profession, the dietitian's role remains underdeveloped and fragmented across Africa. More than 60% of African countries do not have academic programs for training dietitians and the “registered dietitian” title is not protected by law in many African countries.^1^^,^^7^ Most African countries, including Malawi, lack dietetics professional associations, have no formal registration requirements for dietitians and dietetics programs, nor any codes of ethics or a well-articulated scope of practice.^1^^,^^2^^,^^7^ All these factors are important for building the integrity of the profession.

Despite a globally observed exponential growth in the dietetics profession, the dietitian's role remains underdeveloped and fragmented across Africa.

Moreover, most nutrition professionals in Africa receive education and skills largely focused on public health and community nutrition. This nutritionist cadre is critical and has been at the forefront of combating undernutrition, which persists as a major contributor to morbidity and mortality in sub-Saharan Africa. However, management of malnutrition in clinical settings, whose etiologies and treatment are often specific to underlying disease processes, which is just as critical and requires strong integration of medicine and nutrition, is largely underdeveloped. Dietitians specialize in the integration of medical nutrition therapy in individualized patient management plans, while also being adept in working in community-based/public health interventions. Considering the need for developing the dietetics profession, we share the experience and lessons learned in developing and implementing the first dietetics training program in Malawi.

## HEALTH AND NUTRITION SITUATION IN MALAWI

Malawi is a low-income country with a population of approximately 17 million people, 82% of whom are rural and have a national life expectancy of 61 years.^8^^,^^9^ Malawi's health infrastructure faces chronic and severe shortages of health care professionals, basic equipment, and medications, rendering it inadequate for the needs of the population.^10^ Clinical nutrition services are limited, as shown by low rates of screening, diagnosis, and prescription of nutrition therapy interventions in hospitals, particularly for nonpediatric populations. Up to 40% of clinicians use intravenous dextrose as the sole nutrition supplement as opposed to nutritionally complete enteral or parenteral formulations for critically ill patients.^11^ These patients present with severe and complex metabolic derangements that require aggressive and often specialized nutrition support.^6^^,^^11^ In a small study of 25 patients, the average rate of malnutrition in hospitalized surgical patients in Malawi was 80%, which is double the world average.^12^^,^^13^

Concurrently, Malawi's burden of undernutrition in communities remains high, with 37% of children aged under 5 years stunted and 12% underweight.^14^ Noteworthy is that Malawi has made significant progress in reducing the rates of stunting by more than 10 percentage points in the last 10 years.^14^ Micronutrient malnutrition is also a major public health concern, with 28% of preschool children and 15% of women having iron deficiency and 60% of women, men, and children having zinc deficiency.^15^ While infectious diseases such as HIV/AIDS, malaria, and diarrheal diseases are the leading causes of morbidity and mortality in Malawi,^16^ the picture is changing with an increase in diet-related noncommunicable diseases (NCDs), such as cardiovascular diseases, certain cancers, and diabetes mellitus.

Driven by unhealthy diets/lifestyles and overweight/obesity, NCDs are now the leading cause of death globally and show no signs of abating, especially in low and middle-income countries.^17^ In Malawi, 28% of adult women are overweight or obese, and the rate nearly doubles (44%) in urban areas compared to the national average.^8^^,^^18^ The prevalence of hypertension is estimated at 33% of the adult population, 8.7% have abnormal lipid profiles, and diabetes affects about 6% of the population.^8^^,^^18^

Having access to adequate food for the promotion of good health and self-sufficiency is a human right and a central feature of key national documents, including the “National Multi-Sector Nutrition Policy 2018–2022” and its accompanying “National Multisector Nutrition Strategic Plan.”^19^ Of the 8 strategic goals outlined in both national documents, dietetic interventions respond directly to 4: prevention of undernutrition, treatment and control of acute malnutrition, prevention and management of overweight and nutrition-related NCDs, and enhancement of nutrition education.

We outline the process to determine the need for the program along with discrete steps in the development and implementation of the program. We also identify lessons learned and future directions for the continued growth of dietetics training and the profession in Malawi.

## ASSESSING THE NEED FOR DIETITIANS IN MALAWI

**FIGURE 1:**
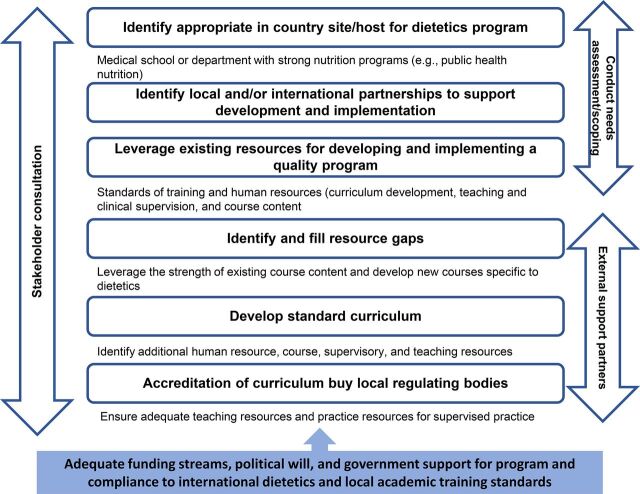
Steps in Developing Malawi's First Dietetics Training Program

We conducted several scoping exercises aimed at understanding the context in Malawi and identifying resources available for running a quality dietetics training program. We identified institutional strengths to implement quality training and key stakeholders that included government officials, whose support was essential to implement the program and promote dietetics practice (Figure 1).

In 2012, we undertook a needs assessment that highlighted the lack of dietitians in Malawi and determined gaps in the health care team that needed to be filled.^21^ We surveyed university leadership and faculty, Ministry of Health staff and leadership, United Nations agencies, and other nongovernmental organizations. Survey respondents stated that the lack of clinical nutrition experts left major gaps in diet therapy, nutrition education, and technical guidance in nutrition programs in Malawi. Respondents all strongly agreed upon the critical need to address the lack of knowledge and skills in nutrition management of diet-related NCDs and critically ill, hospitalized patients, resulting in the development of a program with a clinically focused curriculum that emphasized hospital-based nutrition support interventions and diet-related NCD prevention and management. The program focused on a cadre with research, professionalism, and leadership competencies to generate evidence and drive the development of clinical nutrition services and quality improvement in Malawi.

We undertook a needs assessment that highlighted the lack of dietitians in Malawi and determined what gaps would be filled through the development of this cadre of health care workers.

## CURRICULUM DEVELOPMENT

**FIGURE 2:**
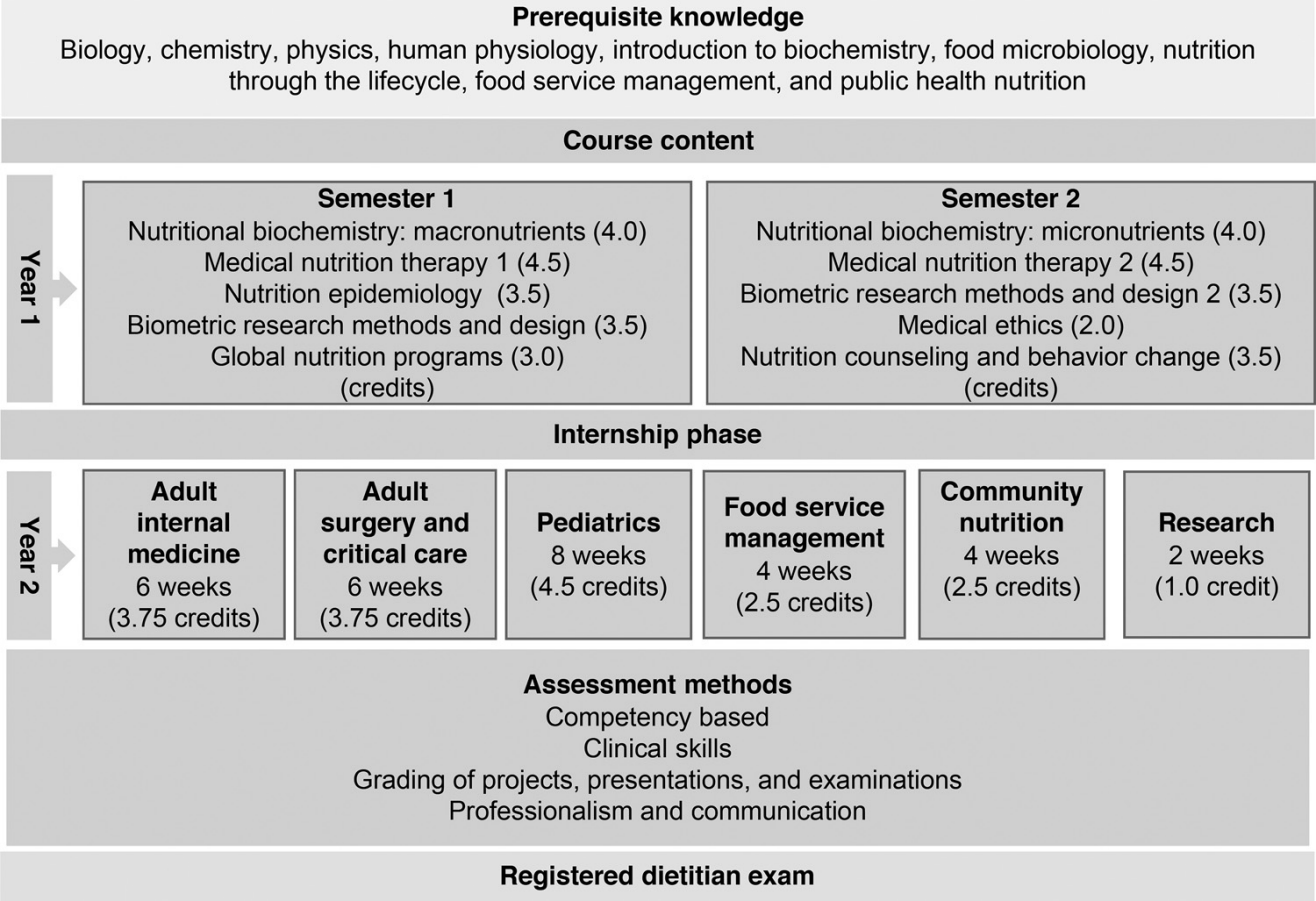
Structure of Postgraduate Diploma in Clinical Dietetics in Malawi

We reviewed the existing Bachelor of Science degree in nutrition and food science at Lilongwe University of Agriculture and Natural Resources (LUANAR) to determine areas where course content merged with the minimum requirements for dietetics and gaps that needed to be filled by the dietetics curriculum. The areas of convergence became the prerequisite requirements for entry into dietetics training (Figure 2). In some instances, new competency-based course content was developed, and in others, existing postgraduate courses were adapted to meet dietetic competencies. Resources for the new course content were modeled from existing well-established standards from the United States of America, South Africa, Kenya, and the International Confederation of Dietitians (ICD) that prescribe minimum course content, length, scope, and competency standards.^19^^–^^21^ Course content was further tailored to respond directly to the needs of Malawi, articulated at the national policy level and through the needs assessment.^22^^,^^23^

## THE FIRST MALAWIAN DIETETICS PROGRAM

The first Malawian clinical dietetics program was created in collaboration with LUANAR, the Feed the Future Innovation Lab for Nutrition based at Tufts University, and the Kamuzu University of Health Sciences (KUHeS) in Blantyre, Malawi (formerly known as the University of Malawi, College of Medicine). The Malawi Mission of the United States Agency for International Development (USAID Malawi) provided funding for the program.

The program equips students with knowledge, attitudes, and skills in nutrition support, health promotion, and disease prevention at the community and health facility levels. During the first 12 months of didactic instruction, students take courses in biomedical sciences, medical nutrition therapy, human behavioral sciences, and research methods (Figure 2). In the subsequent 8 months, students fulfill practical internship requirements in clinical nutrition, community nutrition, and food service management. After completing the 20-month program, graduates receive a postgraduate diploma in clinical dietetics and are encouraged to advance to a recently introduced master's degree, which together take at least 32 months to complete. The criteria for program admission include a bachelor's degree in nutrition or related biomedical science and a minimum grade point average in prerequisite courses (Figure 2). These criteria and requirements are in line with international training standards^20^ for dietitians, with the intent of increasing the rigor of training. All students must pass a final qualifying exam to be eligible for registration to practice.

## PROGRAM IMPLEMENTATION

Both the diploma and master's programs were approved by the LUANAR senate (as the host institution of the program), the Malawi Medical Council, and the National Council for Higher Education. In April 2016, the first cohort of 6 students was admitted; a second cohort of 10 students was admitted in 2018. At the time of writing this article, 10 students have completed all requirements for registration as dietitians. Of the 16 students in the first and second cohorts, 6 either failed to meet pass requirements or dropped out of the program before completion.

Curriculum implementation relied heavily on leveraging the strengths of a strategic partnership between LUANAR, KUHeS, and Tufts University. As an agricultural university with a vibrant and nationally recognized department of human nutrition, LUANAR's expertise and postgraduate courses in food science, public health nutrition, and nutrition epidemiology were adapted to meet dietetics core competencies. Tufts University and KUHeS provided teaching support in core dietetics courses, medical nutrition therapy, nutrition counseling and behavior change, and nutritional biochemistry. Tufts provided technical leadership and ensured the benchmarking of the program to quality standards. KUHeS was also central in providing the platform and supervision for clinical practice.

Considering the lack of dietetics expertise and supervision capacity in Malawi, we identified 2 positions, supervising dietitian and clinical coordinator, that were critical for the daily management of the program, teaching, and supervision. A content expert in clinical dietetics, the supervising dietitian had an advanced degree in dietetics and extensive clinical experience. The clinical coordinator, a Malawian trained medical doctor, brought medical expertise coupled with knowledge and experience on Malawian health care delivery.

Considering the lack of dietetics expertise and supervision capacity in Malawi, we identified 2 positions, supervising dietitian and clinical coordinator, that were critical for the daily management of the program, teaching, and supervision.

An evaluation of dietetics practical training needs against the available resources revealed the lack of adequate critical care nutrition resources (enteral and parenteral nutrition support). In light of this need, the University of Cape Town (UCT) hosted Malawian students for a 6-week exchange program in critical care and surgical nutrition support. The exchange program was critical in building capacity and fulfilling competencies in critical care nutrition support and surgery for the Malawian students, who would have otherwise not been exposed to these resources.

Approaches to providing adequate supervision focused on self-directed methods of learning, which included problem-based learning, peer supervision, and role emerging placements. Because of the lack of dietitians to shadow in Malawian hospitals, role emerging placements were particularly useful, and have gained global traction in allied health care professional training as a viable solution for balancing competing demands of providing quality practical training with limitations in supervisor coverage.^24^^,^^25^ In the latter rotations of the program, students were paired and placed in situations where they were the only dietitians providing services and often reporting back to a nondietitian on-site supervisors (e.g., doctors, nurses, and nutritionists), with the supervising dietitian providing oversight and support. This approach proved useful in establishing input for dietetic services and met a recognized need for dietetic services.

## STAKEHOLDER ENGAGEMENT AND ADVOCACY

While developing the program with various stakeholders, we encountered some barriers that included inadequate knowledge of dietitians' roles, lack of recognition, and low acceptance of the dietitian's role in health care. We actively worked on promoting the new dietetics cadre at the government policy level and to fellow clinicians in hospitals through frequent stakeholder engagements that took many forms.

These stakeholder engagements included aligning the program's aims and objectives to the strategic nutrition and health objectives of the Malawian government through multiple consultations with the government at key points, starting with scoping visits, the needs assessment, and regular program updates to senior Ministry of Health officials. During this process, we identified a need to create demand for dietitians as part of the health care teams. We worked closely with the Department of Clinical Services to establish the first clinical dietetics posts in civil service.

To immerse key government decision makers in clinical dietitians' roles, we invited senior government officials, comprising directors of clinical services, nutrition, and human resources, to visit both a Malawian hospital and UCT teaching hospitals, where students completed their critical care and surgery rotations. This “show, tell, and involve” strategy was instrumental in fast tracking a roadmap for recruiting and integrating dietitians into Malawian clinical services. Although the full integration of dietitians is a long-term goal, the government has shown strong commitment by creating posts and hiring the first graduate registered dietitians. The visits also helped orient the stakeholders on resources needed for optimal practice. We supported the government with mapping the resources needed for optimal practice including procurement needs for specialized enteral and parenteral nutrition support and the development of national hospital food service guidelines. For the first time, these guidelines quantify and standardize the nutritional content of meals served to patients and have therapeutic adaptations for common conditions, such as diabetes and chronic kidney failure, in hospitalized patients.

We used a “show, tell, and involve” strategy with key government decision makers to fast track a roadmap for recruiting and integrating dietitians into Malawian clinical services.

To further address the barrier of inadequate knowledge of dietitians' roles in clinical care, we facilitated clinical nutrition training for doctors and nurses at 2 major referral hospitals in Malawi. Thirty-five critical care nurses and doctors attended a hands-on short course in enteral and parenteral nutrition support administration. This was a precursor to the introduction of ready-to-hang, pump-assisted enteral nutrition administration at 2 of the largest critical care units in Malawi.

## LESSONS LEARNED

The need for dietetic professionals to join health care teams to combat both forms of malnutrition and tackle the rising prevalence of NCDs in developing countries is urgent. Since 60% of African countries were in a similar situation as Malawi before the introduction of the dietetics program, we believe that several lessons can be drawn from the experience in Malawi that may apply to other countries.

### Engage Stakeholders Early and Often

First, engaging with stakeholders from the beginning at both the policy and implementation level garnered government support and drove demand for dietitians in government health services. Sustained engagement efforts with key government stakeholders including sharing regular updates/progress reports, sensitizing them to dietitian roles, and involving senior government management in training students and career development, was crucial in creating a program that developed competencies and skills that responded directly to the country's needs. This response, in turn, was a key factor in gaining high-level government support.

### Develop Career Opportunities

The creation of the first-ever posts for dietitians and subsequent deployment of both cohorts of graduates within months of their graduation was a major achievement considering that more than 50% of existing health care professional posts in Malawi are vacant.^26^ All 10 of the graduates are employed by the government and now cover all 4 tertiary hospitals in the country. We believe that securing employment prospects and career paths for dietitians early in the training program has been and will continue to be a crucial enticing factor for recruiting students, evidenced by the 3-fold increase in the number of applications from the first to the second cohort.

We believe that securing employment prospects and career paths for dietitians early in the training program has been and will continue to be a crucial enticing factor for recruiting students.

### Build Professional Networks and Partnerships

At the implementation level, we found that continuous benchmarking of courses, rotation sites, and competencies was critical in identifying gaps in dietetics training and developing quick remedies for such gaps. For example, the UCT exchange program was a stop-gap remedy that improved the quality of critical care nutrition support training to a level that was not possible in Malawi given the available resources levels there.

Since there were few dietitians in Malawi, the quality of training and exposure relied heavily on leveraging the strengths of partnerships and multiple stakeholder engagement. The medical school partnership was crucial to implementing clinical rotations and gaining quick acceptance and recognition of dietitians in the hospital. The UCT exchange program exposure was also very valuable for Malawian students and preceptors. Students gained confidence in resources for nutrition support that were not available in Malawi and were exposed to an established clinical nutrition service operation, which can be modeled in Malawi. Malawian preceptors also learned about supervision and assessment of students in practical rotations, leading to improved quality in training.

This also highlights the importance of building strong networks with regional dietetics experts who assisted with strengthening our program by vetting examinations and performing a curriculum review. The continued fostering of relationships with regional experts has also opened doors for institutional collaborations in capacity building and research. Two program graduates have completed master's degrees in nutrition with the North-West University, South Africa, for example. In addition, networking with local Malawian dietitians also contributed vastly to improved preceptor support. The preceptors were role models to students and brought extensive practical experience in the Malawian context. Another successful strategy for dietetics clinical practice was a series of dietetics-orientated capacity-building workshops and seminars for other health care professionals. These efforts raised the awareness of the role of the dietitian and advanced multidisciplinary collaboration.

### Build Knowledge Capacity for Dietetics Among Nutritionists

This program was built on maximizing existing capacity for nutrition training by reviewing, adopting, and adapting existing nutrition coursework and matching with dietetics competencies, then filling the gaps with new course content. The recruitment of qualified nutritionists and people with biomedical backgrounds meant that a significant portion of dietetics coursework needs and competencies had already been fulfilled in the student's undergraduate degrees. This led to a shorter program and fast-tracked graduate output. However, through early and continuous evaluation of student performance, we observed many students, particularly those with a nutrition background, struggled to transition from their undergraduate biomedical science knowledge base to the expectations of the dietetics program. This may explain the 37.5% average failure rate observed in both cohorts. Hence, we designed an 8-week “bridging course” to mitigate this gap in nutritional biochemistry, nutrition in the lifecycle, and medical terminology. We hoped that this would lead to a smoother transition between the student's prior knowledge and the rigor of the dietetics program. This may be applicable in other countries, especially in Africa where there are more nutritionist training programs than dietetics.

### Create Alternative Models for Supervision Support

There remained an acute shortage of supervision capacity for students during clinical placements/rotations, a high supervisor-to-student ratio, and no established role for dietitians in clinical placements. We had anticipated this problem given the limitations in dietetics practice in Malawi. Nonetheless, this shortage posed a serious threat to quality training that will likely continue until a critical mass of dietetics educators/instructors exists. We used a role emerging placements model, which works ideally in places where there are no structures and roles for dietitians,^27^ and were highly successful in empowering students, building confidence in communication, promoting clinical reasoning, and improving their sense of their own role in multidisciplinary teams.^24^^,^^25^^.^^27^ Under supervision, students were encouraged to directly interact with the medical teams on patient management by contributing to medical rounds and documenting directly in clinical notes after approval by supervisors.

We used a role emerging placements model, which works ideally in places where there are no structures and roles for dietitians.

## CONCLUSION

A strong case has been made for registered dietitians as an essential piece in solving the nutrition challenges in Malawi. We describe how capacity for the dietetics professional practice has been built thus far. We believe that our case study can be extended to other developing countries that are undergoing similar epidemiological transitions with similar resource constraints impeding the quality of health care professional training.

A major priority for the program going forward is to increase graduate output and build a critical mass of dietitians to sustain the practice. To that end, LUANAR has secured an additional 5-year USAID grant to provide scholarship support for about 25 more students and sustain the program for an additional 5 years. Through this grant, LUANAR will prioritize training dietitian educators through mentorship with regional and international experts. There is a need to secure more sustainable sources of funding from the government and diversify sources of funding by securing research grants and competitive tuition rates.

Efforts to build the national research agenda are underway with 90% of the graduates of the program now pursuing Masters in Dietetics degrees and undertaking groundbreaking research on nutrition outcomes in clinical care. The new grant is also set to bolster the research capacity through training more master's students and funding research. Evidence will ultimately inform practice, improve the quality of care, and justify the role of dietitians in Malawi.

Important steps in promoting professional dietetics practice made thus far have included advocacy, forming a dietetic association, drafting regulatory guidelines that define the scope of dietetics practice and have credentialing requirements for both individuals and training institutions, and engaging national regulators for enacting the guidelines and drafting practice guidelines. These are all structural priorities that protect the registered dietitian title and the public from imposters. Future priorities should focus on strengthening regulation of practice and the association as key bodies promoting practice.

The development and implementation of the first academic program for dietitians marks the genesis of the profession in Malawi. However, sustained investment in the expansion and improvement of practice conditions is needed for dietitians to realize their full potential.

## Notes

**Peer Reviewed**
